# The importance of face-shape masculinity for perceptions of male dominance depends on study design

**DOI:** 10.1038/s41598-023-39912-x

**Published:** 2023-08-03

**Authors:** Junzhi Dong, Kathlyne Leger, Victor K. M. Shiramizu, Urszula M. Marcinkowska, Anthony J. Lee, Benedict C. Jones

**Affiliations:** 1 Department of Psychological Sciences and Health, University of Strathclyde, Glasgow, UK; 2 Institute of Public Health, Jagiellonian University Medical College, Kraków, Poland; 3 Division of Psychology, University of Stirling, Stirling, UK

**Keywords:** Psychology, Human behaviour

## Abstract

Dominance perceptions play an important role in social interactions. Although many researchers have proposed that shape masculinity is an important facial cue for dominance perceptions, evidence for this claim has come almost exclusively from studies that assessed perceptions of experimentally manipulated faces using forced-choice paradigms. Consequently, we investigated the role of masculine shape characteristics in perceptions of men’s facial dominance (1) when shape-manipulated stimuli were presented in a forced-choice paradigm and (2) when unmanipulated face images were rated for dominance and shape masculinity was measured from face images. Although we observed large effects of masculinity on dominance perceptions when we used the forced-choice method (Cohen’s ds = 2.51 and 3.28), the effect of masculinity on dominance perceptions was considerably smaller when unmanipulated face images were rated and shape masculinity measured from face images (Cohen’s ds = 0.44 and 0.62). This pattern was observed when faces were rated separately for physical dominance, social dominance, and masculinity, and was seen for two different sets of stimuli. Collectively, these results suggest that shape masculinity may not be a particularly important cue for dominance perceptions when faces vary simultaneously on multiple dimensions, as is the case during everyday social interactions.

## Introduction

Dominance perceptions of men’s faces are widely assumed to reflect impressions of men’s ability to obtain and hold resources (see^1^ for a review). Indeed, dominance ratings of men’s faces are positively correlated with measures of their actual physical strength and formidability^2–4^. Most research on the physical characteristics of faces that influence perceptions of men’s dominance have investigated the possible effect of masculine shape characteristics e.g.,^5–10^. These studies have concluded that masculine face-shapes have a large positive effect on perceptions of men’s dominance e.g.,^5–10^.

Studies that have argued that masculine shape characteristics have large positive effects on perceptions of men’s dominance have investigated this issue by experimentally increasing or decreasing masculine shape characteristics in face images using computer graphic methods and asking participants to indicate which version of the face looks more dominant e.g.,^5–10^. However, results of other recent studies suggest that this ‘two-alternative forced-choice method’ may be problematic. For example, studies of facial-attractiveness judgments have shown that results obtained from the two-alternative forced-choice method are qualitatively different to those obtained when natural (i.e., unmanipulated) faces are rated for attractiveness and shape characteristics are measured objectively from faces^11–13^. These studies found that the large effects observed for shape preferences assessed by presenting shape manipulated stimuli within a two-alternative forced-choice paradigm are not typically evident when unmanipulated faces are rated and shape characteristics measured from the face images^11–13^. Findings such as these suggest that results obtained when shape manipulated stimuli are presented within a two-alternative forced-choice paradigm are not necessarily instructive regarding the information people use to judge faces under more natural viewing conditions (i.e., when faces vary simultaneously on multiple dimensions^11–14^). In other words, while results for the forced-choice paradigm using manipulated stimuli suggest that people *can* use the manipulated characteristic to make attractiveness judgments, results from tasks in which unmanipulated faces were rated individually suggest people do not necessarily actually use those characteristics to make attractiveness judgments when other information is available, as would be the case during everyday social interactions (for a detailed discussion of the importance of this distinction between ‘can use’ and ‘do use’ in studies of the mapping of social judgments to physical characteristics, see^15^). In a similar vein, results of other studies investigating the suitability of the forced-choice method for studying attractiveness judgments suggest that the forced-choice method assesses face discrimination ability, rather than preferences, per se^16,17^. These results raise further concerns about the extent to which findings from studies using the forced-choice method generalise to investigations using more naturalistic paradigms.

Research comparing the effects of shape characteristics on social judgments of faces assessed when manipulated stimuli were presented within a two-alternative forced-choice paradigm and when image-rating methods were used and shape characteristics measured from natural face images has investigated attractiveness judgments only^11,12^. No previous study has investigated this issue for dominance judgments and only one study has investigated possible relationships between dominance ratings of unmanipulated face images and measured face-shape masculinity. This latter study reported inconsistent results for samples of African and European male faces^18^. Consequently, we compared the effects of masculine face-shape characteristics on perceptions of men’s dominance when dominance perceptions of shape-manipulated images were assessed using the two-alternative forced-choice method and when dominance perceptions of natural (i.e., unmanipulated) faces were assessed using an image-rating paradigm and shape masculinity measured from the face images (Study 1).

In Study 2, we extended the procedure used in Study 1 to investigate the effects of face-shape masculinity on perceptions of two specific components of men’s dominance; physical dominance and social dominance. We carried out this second study because some previous research on dominance perceptions has broken dominance perceptions down into these two components, which are hypothesised to be different routes through which individuals can obtain and retain resources^10,19^. Finally, to test whether observed effects generalized across stimulus sets, in Study 3, we replicated Study 1 using a different set of face images. In Study 4, we tested whether the pattern of results observed for dominance judgments also occurred for masculinity judgments.

## Study 1

In Study 1, we investigated the effects of face-shape masculinity on perceptions of men’s dominance when (1) masculine shape characteristics were experimentally manipulated in images of men’s faces and dominance perceptions were assessed using the two-alternative forced-choice method and (2) when face-shape masculinity was measured from unmanipulated face images and individual men’s faces were rated for dominance using a rating paradigm.

## Methods

### Ethics

Procedures used in all four studies were approved by the School of Psychological Sciences and Health (University of Strathclyde) Ethics Commitee, all work was undertaken in accordance with the Declaration of Helsinki, and all participants provided informed consent.

### Participants

Fifty heterosexual men (mean age = 39.80 years, SD = 15.40 years) and 50 heterosexual women (mean age = 38.48 years, SD = 13.84 years) provided dominance perceptions in this online study. All participants were recruited through Prolific.

### Stimuli

Stimuli were manufactured from an open-access face-image database^20^ depicting images of 50 young adult white men (mean age = 24.2 years, SD = 3.99 years) and 50 young adult white women (mean age = 24.3 years, SD = 4.01 years). Participants posed with neutral expressions and gaze directed at the camera. Images were standardised on pupil position and clothing was masked.

**Figure 1:**
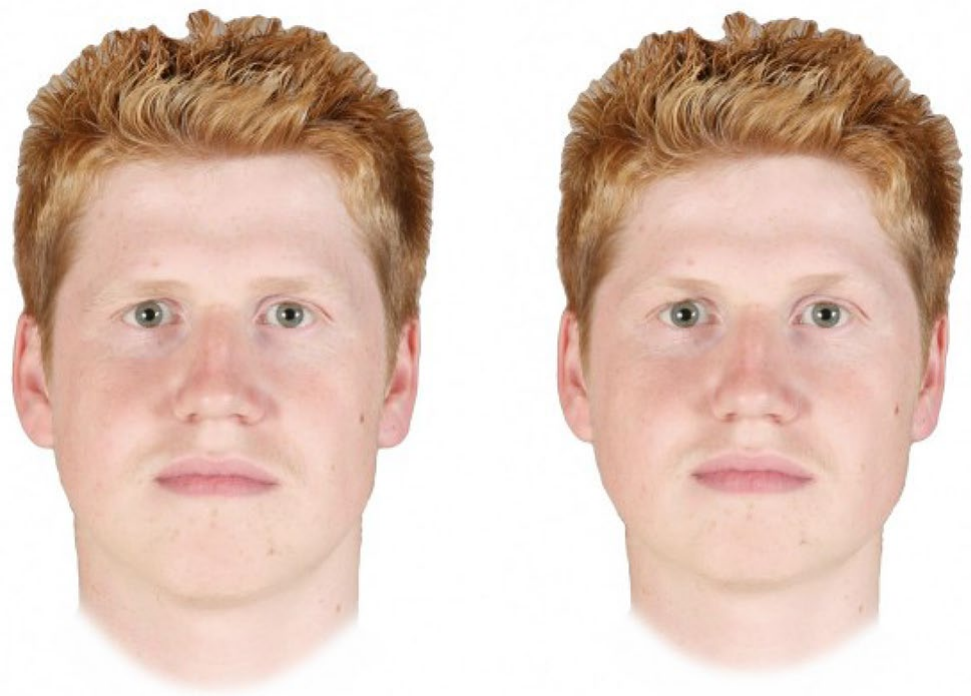
Examples of masculinised (left) and feminised (right) versions of male face images used in the forced-choice task in Study 1 and Study 2.

Masculinised versions of each of the 50 male face images were manufactured by adding 50% of the differences between the average shape for the 50 male face images and the average shape for the 50 female face images to each individual male face. Feminised versions of each of the 50 male face images were manufactured by subtracting 50% of differences between the average shape for the 50 male face images and the average shape for the 50 female face images from each individual male face. This process created fifty pairs of male face images, each pair depicting two images of the same individual that differed in sexually dimorphic shape characteristics only. Examples of the masculinised and feminised versions of the face images are shown in Fig. 1. The computer-graphic methods used to carry out these shape manipulations are described in Perrett et al.^8^ and have been widely used to manufacture face stimuli in studies of the links between sexually dimorphic shape characteristics and dominance perceptions using experimentally manipulated face images^5–10^. Image manipulations were carried out using Webmorph software^21^. All stimuli are publicly available on the Open Science Framework (https://osf.io/a3947/).

### Procedure

Each participant completed two dominance-perception tasks (the forced-choice task and the image-rating task). The order in which they completed these two tasks was fully randomised. In the forced-choice task, participants were shown the fifty pairs of masculinised and feminised male faces and were instructed to click on the person in each pair that looked more dominant. Trial order and the side of the screen any given image was presented were fully randomised. In the image-rating task, participants were shown the fifty original (i.e., unmanipulated) male faces and were instructed to rate the dominance of the person shown using a 1 (much less dominant than average) to 7 (much more dominant than average) scale. Trial order was fully randomised. The study was run using Experimentum software^22^.

### Measuring face-shape masculinity

Face-shape masculinity was objectively assessed for each of the original male face images using the facefuns package^23^ in R^24^. This method has been used to assess face-shape masculinity in many previous studies^25–27^. Shape components were first derived from Principal Component Analysis (PCA) of 132 Procrustes-aligned landmark points (see^25^ for a diagram showing these facial landmarks) on each of the 50 faces. Masculinity scores were then calculated for each image using a vector analysis method^25–27^. This method uses the shape principal components to locate each face on a female-male continuum, defined by calculating the average shape information for the 50 female faces and the average shape information for the 50 male faces. Masculinity scores were then derived by projecting each image onto this female-male vector. Higher scores indicate more masculine face shapes. No shape-masculinity scores were more than three standard deviations from the mean (i.e., there were no extreme scores).

## Results

### Main analyses

All analyses were carried out using R^24^, with the packages kableExtra 1.3.4^28^, jtools 2.2.3^29^, psych 2.2.5^30^, lsr 0.5.2^31^, tidyverse 1.3.1^32^, effectsize 0.7.0^33^. All data, full outputs, and analysis code are publicly available on the Open Science Framework (https://osf.io/7tw4y/).

First, we calculated the proportion of trials on which each participant chose the masculinised version as the more dominant on the forced-choice task (M = 0.86, SD = 0.14). Choices on the forced-choice task showed high reliability across trials (Cronbach’s alpha = 0.90). Next, we used a one-sample t-test to compare these scores with what would be expected by chance (i.e., 0.5). This analysis showed that participants chose the masculinised version significantly more often than would be expected by chance alone (t = 25.15, df = 99, p < 0.001, Cohen’s d = 2.51).

Second, we calculated the mean dominance rating given to each face on the image-rating task. Inter-rater agreement for these ratings was high (Cronbach’s alpha = 0.98). Mean dominance ratings and face-shape masculinity were not significantly correlated (r = 0.21, N = 50, p = 0.136). Converting the r value to Cohen’s d (Cohen’s d = 0.44) showed the effect was considerably smaller than (i.e., less than a fifth the size of) the large effect size seen in the forced-choice task.

### Additional analyses

In addition to the analyses described above, we also analysed responses using mixed effects models. Individual assessments of individual stimuli (rather than mean scores) served as the dependent variable in these analyses. These analyses were carried out using R^24^, with the package lmerTest 3.1-3^34^, and allow variation across both raters and stimuli to be modelled. Analysis code and full results for these analyses are given on the Open Science Framework (https://osf.io/7tw4y/). Results were consistent with those of our initial analyses (i.e., they also showed a large positive and significant effect of masculinity for the forced-choice task and a substantially smaller non-significant effect of masculinity for the image-rating task).

## Study 2

In Study 2 we replicated the procedure used in Study 1. However, in Study 2 participants were randomly allocated to assess the stimuli for either social dominance or physical dominance. Previous studies of facial dominance using the two-alternative forced-choice paradigm and manipulated stimuli found that face-shape masculinity had a larger positive effect on perceptions of men’s physical dominance than it did on perceptions of men’s social dominance (e.g., Watkins et al.^10^).

## Methods

Stimuli, testing procedures, participant recruitment procedure, and measurement of face-shape masculinity were identical to those used in Study 1, except that participants (101 men, 97 women, 1 who did not identify as male or female; mean age = 39.14 years, SD = 14.58 years) were randomly allocated to assess men’s faces on the forced-choice and image-rating tasks for either physical dominance (102 participants) or social dominance (97 participants). Following previous studies^10,19^, physically dominant individuals were described as individuals who would be likely to win a fistfight with another person of the same sex, while socially dominant individuals were described as those who tell other people what to do, are respected, influential, and often a leader. As in previous studies of the putative effects of masculinity on social and physical dominance^10,19^, these definitions were provided for participants.

## Results

Participant responses were analysed in the same way as in Study 1. Assessments of men’s physical and social dominance were analysed separately. All data, full outputs, and analysis code are publicly available on the Open Science Framework (https://osf.io/7tw4y/).

### Physical dominance

For physical dominance, choices on the forced-choice task showed high reliability across trials (Cronbach’s alpha = 0.90). Analyses of responses on the forced-choice task showed that participants chose the masculinised version significantly more often than would be expected by chance alone (M = 0.88, SD = 0.13, t = 29.07, df = 101, p < 0.001, Cohen’s d = 2.88). Inter-rater agreement for responses on the image-rating task was high (Cronbach’s alpha = 0.98) and mean dominance ratings and face-shape masculinity were not significantly correlated (r = 0.20, N = 50, p = 0.15). Converting this r value to Cohen’s d (Cohen’s d = 0.42) showed the effect was considerably smaller than (approximately a seventh the size of) the effect size seen for the forced-choice task.

### Social dominance

For social dominance, choices on the forced-choice task showed high reliability across trials (Cronbach’s alpha = 0.92). Analyses of responses on the forced-choice task showed that participants chose the masculinised version significantly more often than would be expected by chance alone (M = 0.76, SD = 0.19, t = 13.51, df = 96, p < 0.001, Cohen’s d = 1.37). Note that the effect size here was smaller than (approximately half the size of) the effect size seen in the corresponding analysis for assessments of physical dominance.

Inter-rater agreement for responses on the image-rating task was high (Cronbach’s alpha = 0.96) and mean dominance ratings and face-shape masculinity were not significantly correlated (r = 0.21, N = 50, p = 0.15). Converting the r value to Cohen’s d (Cohen’s d = 0.42) showed the effect was considerably smaller than (less than a third the size of) the effect size seen for the forced-choice task. Note that the effect size here for the image-rating task was very similar to the effect size in the corresponding analysis for assessments of physical dominance. Mean ratings of faces for social and physical dominance were significantly positively correlated (r = 0.78, N = 50, p < 0.001, Cohen’s d = 2.47).

### Additional analyses

In addition to the analyses described above, and following Study 1, we also analysed individual responses using mixed effect models. Full results of these analyses are publicly available on the Open Science Framework (https://osf.io/7tw4y/). Results for the mixed effect models were consistent with those of our initial analyses (i.e., for both physical and social dominance, they showed large positive and significant effects of masculinity for the forced-choice tasks and substantially smaller non-significant effects of masculinity for the image-rating tasks).

## Study 3

To test whether observed results generalised across stimulus sets, we conducted a third study (Study 3). Study 3 was a replication of Study 1 (i.e., compared effect sizes for the effect of manipulated face-shape masculinity on dominance perceptions assessed using the forced-choice task and for the correlation between measured face-shape masculinity and dominance ratings of unmanipulated face images), but used a different set of face images.

## Methods

**Figure 2:**
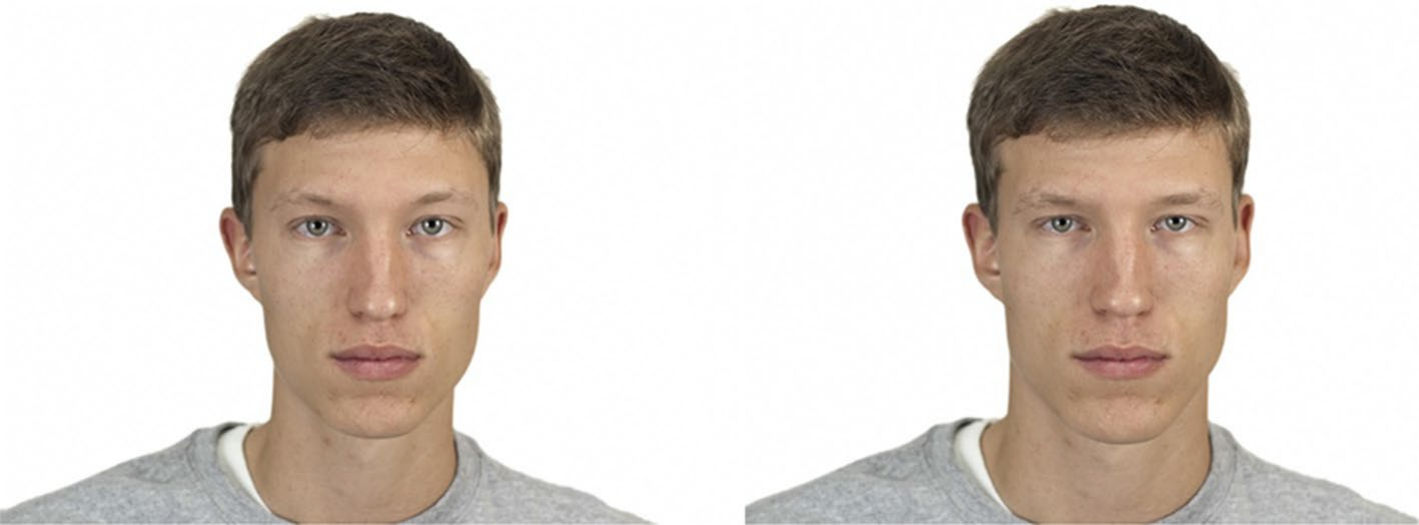
Examples of feminised (left) and masculinised (right) versions of male face images used in the forced-choice task in Study 3.

All aspects of the methods were identical to those employed in Study 1, except that the face stimuli were of 90 white men randomly selected from the Chicago Face Database^35^. For the forced-choice task, masculinised and feminised versions of each face (see Fig. 2) were created using the same procedure described for Study 1. For the image-rating task, the original versions of the 90 male were used, with face-shape masculinity being measured using the same technique described in Study 1. Images of 90 white women from the Chicago Face Database^35^ were also used to measure face-shape masculinity in Study 3. No shape-masculinity scores were more than three standard deviations from the mean (i.e., there were no extreme scores). Face stimuli used in Study 3 are publicly available on the Open Science Framework (https://osf.io/7tw4y/).

Eighty participants (41 men, 39 women, mean age = 36.62 years, SD = 13.43 years) completed the forced-choice task. A different group of 83 participants (40 men, 43 women, mean age = 38.12 years, SD = 12.52 years) completed the image-rating task.

## Results

Responses on the two tasks were analysed in the same way as in Study 1 and Study 2. All data, full outputs, and analysis code are publicly available on the Open Science Framework (https://osf.io/7tw4y/).

Choices on the forced-choice task showed high reliability across trials (Cronbach’s alpha = 0.90). Analyses of responses on the forced-choice task showed that participants chose the masculinised version significantly more often than would be expected by chance alone (M = 0.86, SD = 0.11, t = 29.37, df = 79, p < 0.001, Cohen’s d = 3.28). Inter-rater agreement for responses on the image-rating task was high (Cronbach’s alpha = 0.97) and mean dominance ratings and face-shape masculinity were significantly and positively correlated (r = 0.30, N = 90, p = 0.005). Converting this r value to Cohen’s d (Cohen’s d = 0.62) showed the effect was considerably smaller than (approximately a fifth the size of) the effect size seen for the forced-choice task. Results of the mixed effects models were consistent with those of our initial analyses (i.e., they also showed a large positive and significant effect of masculinity for the forced-choice task and a substantially smaller significant effect of masculinity for the image-rating task). Analysis code and full results for these latter analyses are given on the Open Science Framework (https://osf.io/7tw4y/).

## Study 4

In the previous three studies, we consistently observed large effects of shape masculinity on dominance perceptions assessed using the forced-choice task and manipulated stimuli, while effects were considerably smaller when shape masculinity was measured from natural face images and dominance perceptions were assessed using a rating paradigm. To investigate whether this pattern of results is specific to dominance perceptions or a product of general differences in the two types of tests (forced-choice task using manipulated stimuli versus rating task using natural stimuli), we repeated Study 3, this time asking participants to assess the masculinity of the faces.

## Methods

Methods, procedure, and stimuli were identical to those used in Study 3, except 40 women (mean age = 38.58 years, SD = 12.68 years) and 39 men (mean age = 34.28 years, SD = 10.97 years) were instructed to choose the more masculine face on the forced-choice task and rate the faces for masculinity on the rating task.

## Results

Responses on the two tasks were analysed in the same way as in Studies 1, 2, and 3. All data, full outputs, and analysis code are publicly available on the Open Science Framework (https://osf.io/7tw4y/).

Choices on the forced-choice task showed high reliability across trials (Cronbach’s alpha = 0.95). Analyses of responses on the forced-choice task showed that participants chose the masculinised version significantly more often than would be expected by chance alone (M = 0.86, SD = 0.14, t = 22.72, df = 78, p < 0.001, Cohen’s d = 2.56). Inter-rater agreement for responses on the image-rating task was high (Cronbach’s alpha = 0.96) and mean dominance ratings and face-shape masculinity were significantly and positively correlated (r = 0.26, N = 90, p = 0.013). Converting this r value to Cohen’s d (Cohen’s d = 0.54) showed the effect was considerably smaller than (approximately a fifth the size of) the effect size seen for the forced-choice task. Results of the mixed effects models were consistent with those of our initial analyses (i.e., they also showed a large positive and significant effect of masculinity for the forced-choice task and a substantially smaller significant effect of masculinity for the image-rating task). Analysis code and full results for these latter analyses are given on the Open Science Framework (https://osf.io/7tw4y/).

## Discussion

We compared the effects of masculine face-shape characteristics on perceptions of men’s dominance when (1) dominance perceptions of shape-manipulated images were assessed using the two-alternative forced-choice method and (2) when dominance perceptions were assessed using the image-rating paradigm and shape masculinity measured from face images. Consistent with results of previous studies using the two-alternative forced choice method and shape-manipulated stimuli^5–10^, masculinised versions of faces were perceived to be more dominant than feminised versions. This effect of manipulating face-shape masculinity was seen for general perceptions of dominance (Study 1) and when faces were separately assessed for physical dominance and social dominance (Study 2). Effect sizes were large in all cases (dominance: Cohen’s d = 2.51; physical dominance: Cohen’s d = 2.88; social dominance: Cohen’s d = 1.37) but were smaller for perceptions of social dominance than physical dominance and general dominance. That experimentally manipulating shape masculinity in face images had a smaller effect on perceptions of social dominance than physical dominance is consistent with results of previous work that used the two-alternative forced choice method to compare the effects of face-shape masculinity on perceptions of physical and social dominance (Watkins et al.^10^). In Study 3, using a different set of face images, we also observed a large effect of manipulating face-shape masculinity on dominance perceptions assessed using the forced-choice task (Cohen’s d = 3.28).

Analyses of responses on the rating tasks showed a somewhat different pattern of results to those we obtained using the two-alternative forced-choice method and manipulated stimuli. Most notably, effect sizes for the relationships between dominance ratings and face-shape masculinity were considerably smaller than those obtained using the forced-choice method (dominance: Cohen’s d = 0.44; physical dominance: Cohen’s d = 0.42; social dominance: Cohen’s d = 0.42). By contrast with our results for the forced-choice method, effect sizes for the image-rating task were virtually identical for ratings of social and physical dominance. In Study 3, using a different set of face images, the effect size for the relationship between dominance ratings and face-shape masculinity was also considerably smaller than that obtained using the forced-choice method (Cohen’s d = 0.62). This pattern of results was also seen for masculinity judgments (Study 4), demonstrating the pattern is not specific to dominance perceptions and suggesting that it is a product of general differences in the testing paradigms (forced choice using manipulated stimuli versus rating task using natural stimuli). Moreover, the effect size for the correlation between measured masculinity and masculinity ratings of natural face images was similar to those reported in previous studies that have estimated the effect size for this correlation^13,36^.

Collectively, the results summarised above suggest that, although shape masculinity has large effects on dominance perceptions when it is the only information that differs between the faces being assessed, it plays a much less important role in dominance perceptions of natural (i.e., unmanipulated) faces that vary simultaneously on multiple dimensions (i.e., when other information is available to form impressions of men’s dominance, shape masculinity does not appear to play a particularly important role in dominance perceptions). This pattern of results is noteworthy since, although many researchers have proposed that shape masculinity plays a critical role in dominance perceptions^5–10^, our results suggest that it is a relatively poor predictor of dominance perceptions when dominance is assessed under more natural (i.e., ecologically valid) viewing conditions (i.e., when faces vary simultaneously on multiple dimensions, as they do during everyday social interactions). Importantly, our results were consistent across two different image sets, suggesting they are not specific to a single stimulus set and likely generalise well. Our results for dominance perceptions are consistent with those of previous work on attractiveness judgments^11–13^, which also found that the large effects of manipulated face-shape characteristics evident in studies using forced-choice tasks are typically considerably smaller (and often not significant) when unmanipulated faces were rated for attractiveness and shape characteristics were measured from the face images.

Our results raise the question of what facial information people do use to assess dominance when faces vary simultaneously on multiple dimensions. Many previous studies have reported that dominance ratings of faces are positively correlated with indices of men’s threat potential, such as objective measures of their physical strength and body size^2–4^. Other research has demonstrated the existence of quantifiable shape correlates of physical strength and body size^37,38^ and that these shape parameters can be isolated from sexually dimorphic shape information^39^. It is therefore possible that these shape characteristics, rather than shape masculinity, drive dominance perceptions. Further work comparing the predictive value of these shape characteristics and shape masculinity would resolve this issue. Facial cues of dominance might also not be limited to shape information. Although there has been little work on the role of surface information in dominance judgments, Torrance et al.^40^ found that shape and surface information independently predicted ratings of the dominance of men’s faces and predicted dominance ratings to similar degrees.

In all four of our studies, we compared effect sizes when manipulated stimuli were presented in a forced-choice paradigm and when unmanipulated (i.e., natural) stimuli were presented within a rating paradigm. We carried out this specific comparison because these are by far the two most common approaches used to study social judgments of faces (i.e., it is very unusual for researchers to present unmanipulated stimuli in a forced choice paradigm or present manipulated stimuli in a rating paradigm when investigating the characteristics people use to make social judgments of faces). Nonetheless, we acknowledge here that this comparison confounds testing paradigm (forced-choice task versus rating task) and type of stimuli (manipulated images versus unmanipulated images). Further work is needed to unpack whether the differences observed in our studies (as well as in other work,^11–13^) are due to the testing paradigm, type of stimuli, or a combination of these factors.

In conclusion, across three studies and two different sets of face images, we observed large positive effects of shape masculinity on dominance perceptions when shape information was experimentally manipulated in face images and dominance perceptions assessed using a two-alternative forced-choice paradigm. However, when natural (i.e., unmanipulated) face images were rated for dominance and shape masculinity was objectively measured from face images, effect sizes for the relationships between dominance ratings and shape masculinity were considerably smaller. This pattern of results was also observed when we assessed masculinity, rather than dominance, perceptions (Study 4). Collectively, these results suggest that shape masculinity may not be a particularly important cue for dominance ratings of faces when stimuli vary simultaneously on multiple dimensions (as is the case during everyday social interactions).

## Data Availability

All data and analysis code are publicly available on the Open Science Framework (https://osf.io/7tw4y/). Stimuli for Studies 1 and 2 are publicly available at https://osf.io/a3947/ and stimuli for Studies 3a and 3b are publicly available at https://osf.io/7tw4y/.
